# Fish Parasite Community of Three Lakes with Different Trophic Status in Mecklenburg-Western Pomerania, Germany

**DOI:** 10.1007/s11686-021-00465-6

**Published:** 2021-09-07

**Authors:** Jaydipbhai Suthar, Patrick Unger, Harry W. Palm

**Affiliations:** Aquaculture and Sea-Ranching, Faculty of Agricultural and Environmental Sciences, University of Rostock, Rostock, Germany

**Keywords:** Biological indicators, Parasite communities, Freshwater ecology, Germany

## Abstract

**Purpose:**

The present study investigates the fish parasite fauna from Lake Tollense, Mecklenburg-Western Pomerania, Germany.

**Methods:**

A total of 117 perch (*Perca fluviatilis*), bream (*Abramis brama*) and roach (*Rutilus rutilus*) were sampled for parasites during 2018 and 2019 from Lake Tollense and compared with earlier data from Lake Malchin and Lake Hohen Sprenz in 2011 and 2014, respectively. Parasites were identified based on morphological and molecular characters.

**Results:**

A total of 32 parasite species were isolated from fishes of Lake Tollense, predominated by digeneans. *Diplostomum baeri* was found only in perch while *D. spathacaeum* was isolated from bream and roach. Parasite comparison of similar hosts between lakes revealed highly abundant *Ichthyocotylurus* spp. in perch and bream of Lake Malchin while *Aspidogaster limacoides* was most common in roach from Lake Tollense. Diversity indices of roach showed significant variation between localities. However, NMDS graph revealed separation of the three freshwater habitats based on the parasite fauna of perch, bream and roach.

**Conclusion:**

Based on the multivariate statistical analysis, the three natural inland water bodies could be distinguished based on the parasite communities of perch, bream and roach. The potential to utilise fish parasites as biological indicators in freshwater ecosystem is discussed.

## Introduction

Mecklenburg-Western Pomerania, situated in the North-east of Germany has more than 2000 lakes, most of which are natural and of ice age origin. With a coastline of approx. 2000 km, over 33% of the land area is protected by law and has various unique ecosystems such as Lake Tollense, Malchin and Hohen Sprenz [1]. Lake Tollense is located in the eastern part of the Mecklenburg Lake District, southwest the city of Neubrandenburg, and is one of the largest water bodies in the state (17.9 km^2^) [2]. Lake Malchin is located in the North of the Mecklenburg Lake District with an area of 13.8 km^2^ while Lake Hohen Sprenz is situated south the city of Rostock with a surface area 2.25 km^2^ [2]. According to the Ministry of Agriculture and the Environment of Germany (Data provided upon request; *Seenprogramm MV*), Lake Tollense is classified as mesotrophic 1, Lake Hohen Sprenz eutrophic 2 and Lake Malchin polytrophic 2.

The water bodies harbour diverse fish fauna [2], including the Eurasian perch, *Perca fluviatilis* (L.). This percid fish is adapted to different types of habitats ranging from small to large fresh and brackish water areas [3]. The species undergoes size-related ontogenic shifts in diet, e.g. larvae and juveniles are pelagic zooplankton feeders, switching to benthic invertebrates at intermediate sizes and finally, large sized perch mainly prey on fish [4]. Common bream, *Abramis brama* (L.), and roach, *Rutilus rutilus* (L.) are cyprinids and inhabit fresh- and brackish water [5]. Common bream is a filter-feeder and consumes zooplankton, insects, molluscs, plants and small fish [6, 7]. Roach is omnivorous, feeding on benthic invertebrates, zooplankton, molluscs, plant material and detritus [3, 8]. The two cyprinids are widely distributed in Europe [5] and in the lakes of northern Germany [2, 9].

The status of a freshwater habitat can be described by using parasitic organisms as biological indicators. For example, Valtonen *et al.* [10] linked parasite assemblages in roach and perch to the trophic status of four aquatic habitats in Finland—Lake Peurunka (oligotrophic), Lake Saravesi and Leppävesi (both eutrophic) and Lake Vatia (polluted). Moreover, a high-intensity of trichodinid ciliates was recorded on the longhorn sculpin, *Myxocephalus octodecemspinosus* (Mitchill, 1814) from a location exposed to crude oil pulp and paper mill effluents [11], and Palm and Dobberstein [12] suggested these ciliates as successful sentinels for eutrophication. Galli *et al.* [13] examined chub [*Leuciscus cephalus* L. now *Squalius cephalus* (L.)] from different freshwater localities in Italy and demonstrated that parasite distribution was influenced by the levels of pollution. They observed that the copepod *Lamproglena pulchella* (von Nordmann, 1832) and acanthocephalan *Pomphorhynchus laevis* (Zoega in Müller, 1776) were restricted to the unpolluted and slightly polluted habitats respectively, and digeneans *Asymphylodora tincae* (Modder, 1790) and *Diplostomum spathaceum* (Rudolphi, 1819) were absent in fish from severely polluted site. Palm [14] summarised the use of fish parasites as biological indicators, including the potential to indicate eutrophication and pollution.

Salzman *et al.* [15] attempted to use roach as a biological indicator for habitat size and pollution in anthropogenic-influenced and man-made freshwater habitats in North Rhine-Westphalia, Western Germany. Parasitological investigations of fishes have been well documented in the German coastal waters [16, 17]. However, despite numerous of lakes in this region, relatively few studies have been carried out on freshwater fish parasites in Mecklenburg-Western Pomerania. The most comprehensive study by Pikalov [18] listed 92 parasite species from freshwater fishes: 37 from perch, bream and roach. The purpose of the present study is an analysis of the parasite fauna of perch, bream and roach from three natural freshwater habitats (Lake Tollense, Lake Malchin and Lake Hohen Sprenz) of ice age origin in Mecklenburg-Western Pomerania. The current study was a detailed survey of fish parasites of Lake Tollense. Additionally, data from the other two lakes (by Pikalov) were incorporated to investigate the link between the conditions of the habitat (trophic status) and parasite diversity. The parasite communities are used as biological indicators with respect to the known characteristics of the lakes.

## Materials and Methods

### Sample Site and Collection

In total, 117 specimens of wild freshwater fishes belonging to perch, *P. fluviatilis* (*n* = 40), bream, *A. brama* (*n* = 30) and roach, *R. rutilus* (*n* = 47) were sampled from Lake Tollense, 17.9 km^2^, maximum depth 31.3 m (mean 17.8 m) [2], during 2018–2019. Host fishes (perch = 35, bream = 35 and roach = 35) were collected by Pikalov [18] from Lake Malchin, 13.8 km^2^, maximum depth 10.0 m (mean 2.5), in 2011, and Lake Hohen Sprenz, 2.25 km^2^, maximum depth 17.3 m (mean 7.0) [2] (roach, *n* = 35), also Mecklenburg-Western Pomerania, northern Germany. In 2014, with the original data set analysed and compared with the new samplings. All fishes were captured by local fishermen using fish traps and gillnet. The fishes were stored separately in labelled plastic bags and kept on ice during transport to the laboratory. They were deep frozen (− 18 °C) on arrival at University of Rostock for subsequent parasitological investigation.

### Host Examination and Parasite Collection

In the laboratory, fishes were defrosted at room temperature, measured (total and standard length), internal organs include weight of (total weight, gutted weight, internal organs such as liver, gonads, full stomach and empty stomach), and their sex was determined. Initially, according to the standard procedure of Palm and Bray [19], the external parts of the fishes (e.g. skin, eyes, gills, opercula, fins, buccal and mouth cavity) were studied for ectoparasites. Then, the body cavity was opened and rinsed with saline solution to detect parasites that inhabits the cavities and those attached to the mesenteries or other internal organs. Internal organs such as eyes, heart, liver, stomach, pyloric caeca, intestine, spleen, kidney, swim bladder, gall bladder and gonads were removed, transferred to a separate Petri-dish, washed in 0.9% saline solution and examined for endoparasites. The examination was carried out under the stereomicroscope (Zeiss Stemi 305). Isolated parasites were placed in small glasses containing 0.9% saline solution, cleaned and fixed in ethanol (70% and 99%) for further morphological and molecular identification, respectively.

### Parasite Morphological and Molecular Analyses

Parasite specimens preserved in 70% ethanol were isolated and permanent mounts prepared following standard staining procedures for each taxon [18, 20, 21]. The parasites were identified to the lowest taxa possible with the help of identification keys available in literature.

Selected Aspidogastrean and Digeneans fixed with 70% EtOH were stained with acetic carmine, whereas Acanthocephala and Nematoda were mounted on glycerine for light microscopic examination (BX53). Ectoparasites (Monogenea and Crustacea) were directly examined under light microscope after being isolated from fishes. The body, organs and taxonomically relevant characters were measured for *Aspidogaster limacoides* Diesing, 1834 and *Contracaecum rudolphii* Hartwich, 1964. Monogeneans, Acanthocephalans, Nematoda and Crustacea were identified based on morphological features as provided by Pikalov [18].

For scanning electron microscopy (SEM), preserved specimens of *A*. *limacoides* were dehydrated in an ascending ethanol series and transferred to 100% acetone (twice for 10 min each), critical point dried (Emitech K850, Co. Quorum Technologies LTD, East Sussex), mounted on SEM-carrier with adhesive conductive carbon tape (Co. PLANO, Wetzlar), coated with gold under vacuum (EM SCD 004, Co. BALTEC, Balzers) and analysed using a field emission scanning electron microscope (FE-SEM, MERLIN^®^ VP Compact, Co. Zeiss, Oberkochen) at the Electron Microscopy Centre, University Medicine Rostock.

DNA was extracted from representative specimens (preserved in 99% ethanol) following the standard protocol given in the DNeasy Blood & Tissue kit (Qiagen, Germany).

*Aspidogastrea*: The ribosomal DNA (rDNA) region comprising the ITS-1, 5.8S, ITS-2 and flanking sequences (= ITS+) were amplified with the universal primers BD1 (5′-GTCGTAACAAGGTTTCCGTA-3′) and BD2 (5′-TATGCTTAA(G/A)TTCAGCGGGT-3′). Each PCR reaction was performed in a thermocycler (Biometra, Göttingen, Germany) under the following conditions: initial denaturation at 94 °C for 3 min, followed by 40 cycles of 30 s denaturation at 94 °C, 30 s annealing at 54 °C and 2 min elongation at 72 °C; and a final extension hold at 72 °C for 7 min [22].

*Digenea*: Complete ssrDNA rDNA was amplified using primers WormA (50-GCGAATGGCTCATTAAATCAG-30) and WormB (50-CTTGTTACGACTTTTACTTCC-30) using the following PCR cycling conditions: denature for 2 min at 94 °C, followed by 40 cycles of 30 s at 94 °C, 30 s at 54 °C, 2 min at 72 °C; and 7 min extension at 72 °C [23].

*Diplostomum* spp.: Cox1 region was amplified by using the diplostomid specific PCR primers: Plat-diplo COX1F (forward; 5′-CGTTTRAATTATACGGATCC-3′) and Plat-diplo COX1R (reverse; 5′-AGCATAGTAATMGCAGCAGC-3′). PCR reaction was performed in a thermocycler (Biometra, Gottingen, Germany) under the following conditions: initial denaturation at 95 °C for 3 min followed by 35 cycles (94 °C for 30 s, 58 °C for 30 s and 72 °C for 2 min), and a final extension step at 72 °C for 5 min [24].

*Nematoda/Anisakidae*: The region of rDNA comprising the ITS-1, 5.8S, ITS-2 and flanking sequences (= ITS+) was amplified using the primers Forward TK1 (5′-GGC-AAA-AGT-CGT-AAC-AAG-GT-3′) and Reverse NC2 (5′-TTA-GTT-TCT-TTT-CCT-CCG-CT-3′). PCR cyclic started with an initial denaturation at 95 °C for 1 min, 40 cycles of 94 °C for 45 s (denaturation), 55 °C for 45 s (annealing), 72 °C for 45 s (extension) and a final extension at 72 °C for 10 min [25].

The PCR products were purified as described in Qiagen DNeasy Blood & Tissue Kit. For the sequencing, a mixture of purified PCR product and the above primers were used and sent to sequencing service (Seqlab, Göttingen).

Contiguous sequences were manually edited and assembled using BioEdit 7.2. The resulting sequences were subjected to nucleotide BLAST searches to find the highest matching sequences available in the Genbank (http://www.ncbi.nlm.nih.gov/blast).

### Parasitological and Diversity Parameters

The ecological terms including prevalence (P), mean intensity (MI), intensity (I) and mean abundance (MA) follow Bush *et al.* [26]. The prevalence of the different parasites was graded into core species (> 60%), secondary species (40–60%), satellite species (5–40%) and rare species (< 5%) after Holmes [27]. Analysis of component community parameters included species richness (number of parasite taxa), Shannon index of species diversity (H), evenness index of Pielou (E), Berger-Parker Index (BP) and Simpson Index (S) were calculated [18, 28].

### Statistical Analysis

Non-metric multidimensional scaling (NMDS) was performed with PRIMER 7 in order to visualise differences in the parasite fauna of roach from the three habitats. Prior to the proceeding of analysis, abundance data were square root transformed to reduce the impact of the dominant species on those with low abundance. Analysis of similarities (ANOSIM) was tested with Bray–Curtis similarity index to detect significant differences in the component community of roach in three lakes.

## Results

### Parasite Identification

The parasitological examination of three fish species, *P. fluviatilis*, *A. brama* and *R. rutilus,* from Lake Tollense revealed a total of 32 metazoan parasite species belonging to the Aspidogastrea (1), Digenea (14), Cestoda (5), Monogenea (4), Acanthocephala (2), Nematoda (4) and Crustacea (2). The parasitological terms such as prevalence, intensity, mean intensity, mean abundance and diversity indices together with the infection sites are presented in Table 1. The highest species richness and diversity was observed in *P*. *fluviatilis* and *A*. *brama*. Molecular identification (COX1 region) of *Diplostomum* spp. revealed the following two species: *Diplostomum baeri* Dubois, 1937 in perch (99% similarity to GenBank accession number KM212030) and *D. spathacaeum* in bream and roach, with 100% and 99% similarity to GenBank accession number KR271459 and KR271429, respectively.

**Table 1:** Parasite fauna of three freshwater fishes from Lake Tollense, northern Germany

Parasite species	*Perca fluviatilis*			*Abramis brama*			*Rutilus rutilus*		
N	40			30			47		
	P (%)	mI (I)	mA	P (%)	mI (I)	mA	P (%)	mI (I)	mA
Aspidogastrea									
* Aspidogaster limacoides*	–	–	–	2.86	18.00 (18)	0.51	61.70	9.59 (1–53)	5.91
Digenea									
* Azygia lucii*	5.00	1.00 (1)	0.05	–	–	–	–	–	–
* Bucephalus polymorphus*	15.00	4.67 (1–12)	0.70	–	–	–	–	–	–
* Bunodera luciopercae*	100.00	173.83 (6–540)	173.83	–	–	–	–	–	–
* Diplostomum baeri*	92.50	10.05 (1–32)	9.30	–	–	–	–	–	–
* D. spathacaeum*	–	–	–	76.67	23.52 (1–213)	18.03	76.60	23.03 (1–246)	17.64
* Gorgoderina lufengensis*	–	–	–	6.67	2.00 (1–3)	0.13	–	–	–
* Ichthyocotylurus variegatus*	7.50	1.00 (1)	0.08	–	–	–	–	–	–
* Lissorchis kritskyi*	–	–	–	–	–	–	2.12	5.00 (5)	0.11
Opecoelidae sp.	–	–	–	56.67	41.65 (1–210)	23.60	31.91	7.27 (1–47)	2.32
* Posthodiplostomum brevicaudatum*	15.00	9.17 (1–42)	1.38	–	–	–	–	–	–
* Rhipidocotyle campanula*	5.00	0.50 (1)	0.03	–	–	–	–	–	–
* Prosorhynchoides borealis*	–	–	–	–	–	–	2.12	1.00 (1)	0.02
* Tylodelphys clavata*	95.00	38.18 (3–97)	36.28	26.67	2.38 (1–4)	0.63	93.62	50.41 (3–187)	47.19
* T. podicipina*	2.50	1.00 (1)	0.03	–	–	–	–	–	–
Cestoda									
* Bothriocephalus scorpii*	2.50	1.00 (1)	0.03	–	–	–	–	–	–
* Caryophyllaeus laticeps*	–	–	–	40.00	16.33 (1–59)	6.53	4.26	1.00 (1)	0.04
* Paradilepis scolecina*	–	–	–	53.33	21.06 (1–94)	11.23	17.02	5.88 (1–23)	1.00
* Proteocephalus percae*	2.50	1.00 (1)	0.03	–	–	–	–	–	–
* Triaenophorus nodulosus*	10.00	2.50 (1–5)	0.25	–	–	–	–	–	–
Monogenea									
* Ancyrocephalus percae*	2.50	1.00 (1)	0.03	–	–	–	–	–	–
* Dactylogyrus sphyrna*	–	–	–	30.00	32.78 (2–87)	9.83	89.36	20.76 (1–140)	18.55
* Diplozoan paradoxum*	–	–	–	46.67	3.57 (1–7)	1.67	–	–	–
* Paradiplozoan homoin*	–	–	–	–	–	–	42.55	3.80 (1–9)	1.62
Acanthocephala									
* Acanthocephalus anguillae*	–	–	–	13.33	1.25 (1–2)	0.17	–	–	–
* A. lucii*	7.50	3.00 (1–7)	0.23	–	–	–	–	–	–
Nematoda									
*Camallanus* spp.	100.00	27.00 (3–91)	27.00	–	–	–	–	–	–
*Contracaecum rudolphii*	–	–	–	83.33	53.28 (2–277)	44.40	29.79	15.42 (1–83)	4.51
*Philometra ovata*	–	–	–	6.67	1.50 (1–2)	0.10	–	–	–
*P*. *rischta*	–	–	–	–	–	–	4.26	1.00 (1)	0.04
Crustacea									
*Argulus foliaceus*	2.50	1.00 (1)	0.03	–	–	–	–	–	–
*Ergasilus sieboldi*	–	–	–	33.33	3.00 (1–10)	1.00	8.51	1.25 (1–2)	0.11
Ecological parameters									
Richness	16			13			13		
Shannon Index (H)	0.96			1.75			1.50		
Evenness (E)	0.35			0.68			0.59		
Simpson Index (S)	1.92			4.45			3.33		
Berger-Parker (BP)	0.70			0.38			0.48		

I, intensity; MA, mean abundance; MI, mean intensity; N, number of examined fishes; P, prevalence

For anisakid nematodes, the ITS1-5.8S-ITS2 region of rDNA were generated from the isolated larval stages (L3) and analysis of BLAST showed 97% similarity to *C*. *rudolphii*, GenBank accession number MH778117. Sequences are deposited in Genbank under the accession numbers MW652700 (*D*. *baeri*, perch), MW652701 (*D. spathaceum*, bream), MW652702 (*D. spathaceum*, roach) and MW652707 (*C. rudolphii*, roach).

For Aspidogastrea, one species *A*. *limacoides* was identified based on morphology and molecular methods with highest prevalence in *R*. *rutilus* (61.7%) and lowest in *A*. *brama* (2.8%) [22].

Digenea was the most diverse taxon of which nine species parasitised *P*. *fluviatilis*, five occurred in *R*. *rutilus* while *A. brama* harboured four species. In *P*. *fluviatilis*, the most abundant digeneans were *Bunodera luciopercae* (Müller, 1776) (P = 100.0%; mI = 173.8), *D*. *baeri* (P = 92.5; mI = 10.1) and *Tylodelphys clavata* (van Nordmann, 1832) (P = 95.0%; mI = 38.2). In *A*. *brama*, *D*. *spathacaeum* (P = 76.7%; mI = 23.2) was present in high numbers followed by Opecoelidae sp. (P = 56.7%; mI = 41.7). Metacercariae of *Tylodelphys clavata* (von Nordmann, 1832) (P = 93.6%; mI = 50.4) and *D. spathacaeum* (*P* = 76.6%; mI = 23) were highly abundant in *R*. *rutilus* (see Table 1).

For Cestoda, five species were found, of which *Bothriocephalus scorpii* (Müller, 1776), *Proteocephalus percae* (Müller, 1780) and *Triaenophorus nodulosus* (Pallas, 1781) occurred in *P*. *fluviatilis*. Additional two species (*Caryophyllaeus laticeps* (Pallas, 1781) and *Paradilepis scolecina* (Rudolphi, 1819)) were found in *A*. *brama* and *R*. *rutilus*, but with different prevalence and intensity. Around fifty percent of *A*. *brama* were infected with adult *C. laticeps* and cysts of *P. scolecina*. In *R*. *rutilus*, the prevalence of infection with *P. scolecina* and *C. laticeps* was 17.0% and 4.3%, respectively.

Two adult Acanthocephala were found in the intestine of perch and bream, *Acanthocephalus lucii* (Müller, 1776) in perch and *A. anguillae* (Müller, 1780) in bream.

Three genera of Nematoda were found. Of these, *Camallanus* spp. was most abundant in perch and *C*. *rudolphii* was most abundant in bream. The genus *Philometra* consisted of two species, *Philometra ovata* (Zeder, 1803) reported in bream and *Philometra rischta* (Skrjabin, 1917) found in roach, but both with low prevalence (Table 1).

Four species of Monogenea were found: of these, *Ancyrocephalus percae* (Ergens, 1996) was present only in perch. *Dactylogyrus sphyrna* (Linstow, 1878) occurred in bream and roach with a prevalence of 30.0% and 89.4%, respectively. The remaining species belonged to the family Diplozoidae, with *Diplozoan paradoxum* (von Nordmann, 1832) recorded from bream while *Paradiplozoan homoion* (Bychowsky & Nagibina, 1959) was reported from roach with almost similar prevalence, mean intensity and mean abundance (Table 1). Two different crustacean species, *Argulus foliaceus* (L., 1758) and *Ergasilus sieboldi* (Nordmann, 1832), were found on the gills. Perch was infected with a single specimen of *A. foliaceus*, while *E*. *sieboldi* was reported in bream and roach. *Ergasilus sieboldi* was more prevalent and abundant in bream (P = 33.3%; mI = 3.0) compared to roach (P = 8.5%; mI = 1.2).

### Diversity Parameters

The highest species richness was recorded in perch (16), while bream and roach harboured 13 species each. The highest Shannon diversity was reported in bream (1.74) followed by roach (1.50) and perch (0.96). Accordingly, evenness was highest in bream (0.68) and lowest in perch (0.35). Simpson index and Berger–Parker index recorded highest value in bream (4.45) and perch (0.70), respectively (see Table 1). In perch and bream, the diversity parameters were very similar, while in roach the species diversity highly differed. After excluding the ectoparasites in calculations for roach, the Evenness, Simpson and Berger–Parker indices became very similar between Lake Tollense (0.51, 2.39, 0.60) and Lake Hohen Sprenz (0.57, 2.11, 0.54), respectively, with the Shannon–Wiener index for the endohelminths remaining more different (1.18 vs 0.79). Lake Malchin still showed different diversity parameters compared with the other two lakes (lower values of Shannon–Wiener Index (0.19), Eveness (0.14) and Simpson Index (1.08) and higher value for Berger–Parker dominance (0.96).

To compare the results fromLake Tollense with Lake Malchin and Lake Hohen Sprenz, two other freshwater lakes in the region, the raw data from Pikalov [18] were analysed and calculated for parasitological terms as given in Tables 2 and 3.

**Table 2:** Parasite fauna of perch and bream from Lake Malchin (changed after Pikalov [18])

Parasite species	*Perca fluviatilis*			*Abramis brama*		
N	35			35		
	P (%)	mI (I)	mA	P (%)	mI (I)	mA
Digenea						
*Azygia lucii*	8.57	1.33 (1–2)	0.11	–	–	–
*Bunodera luciopercae*	71.43	4.12 (1–19)	2.94	–	–	–
*Diplostomum baeri*	48.57	5.71 (1–27)	2.77	–	–	–
*D. spathacaeum*	–	–	–	37.14	5.08 (1–199)	1.89
*Ichthyocotylurus variegatus*	94.29	16.12 (1–80)	15.20	–	–	–
*I. platycephalus*	–	–	–	80.00	8.07 (1–41)	6.46
*Sphaerostoma bramae*	–	–	–	8.57	52.33 (4–144)	4.49
*Posthodiplostomum brevicaudatum*	31.43	3.82 (1–15)	1.20	–	–	–
*P. cuticula*	–	–	–	17.14	1.50 (1–3)	0.26
*Tylodelphys clavata*	80.00	62.71 (2–437)	50.17	45.71	13.69 (1–60)	6.26
*T. podicipina*	2.86	1.00 (1)	0.02	–	–	–
Cestoda						
*Caryophyllaeus laticeps*	–	–	–	8.57	1.00 (1)	0.09
*Ligula intestinalis*	–	–	–	2.86	1.00 (1)	0.03
*Paradilepis scolecina*	–	–	–	51.43	4.00 (1–20)	2.06
*Proteocephalus percae*	2.86	1.00 (2)	0.06	–	–	–
*Triaenophorus nodulosus*	68.57	3.17 (1–11)	2.17	–	–	–
Monogenea						
*Ancyrocephalus percae*	17.14	1.33 (1–3)	0.23	–	–	–
*Diplozoan paradoxum*	–	–	–	14.29	1.60 (1–3)	0.23
Acanthocephala						
*Acanthocephalus lucii*	20.00	1.70 (1–4)	0.34	–	–	–
Nematoda						
*Camallanus* spp.	62.86	4.23 (1–12)	2.66	–	–	–
*Contracaecum rudolphii*	–	–	–	88.57	15.23 (1–65)	13.49
*Philometra ovata*	–	–	–	11.43	3.25 (1–9)	0.37
Crustacea						
*Argulus foliaceus*	5.71	1.00 (1)	0.06	14.29	1.20 (1–2)	0.17
*Ergasilus sieboldi*	5.71	1.00 (1)	0.06	77.14	3.89 (1–12)	3.00
Ecological parameters						
Richness	14			13		
Shannon Index (H)	1.19			1.86		
Evenness (E)	0.45			0.73		
Simpson Index (D)	2.19			5.01		
Berger-Parker (BP)	0.64			0.35		

I, intensity; MA, mean abundance; MI, mean intensity; N, number of examined fishes; P, prevalence

**Table 3:** Parasites fauna of Roach in Lake Malchin and Lake Hohen Sprenz (changed after Pikalov [18])

Parasite species	Lake Malchin			Lake Hohen Sprenz		
N	35			35		
	P (%)	mI (I)	mA	P (%)	mI (I)	mA
Digenea						
*Diplostomum spathacaeum*	48.57	5.59 (1–18)	2.71	37.14	1.77 (1–6)	0.66
*Posthodiplostomum cuticula*	57.14	7.0 (1–62)	4.00	5.71	1.00 (1)	0.06
*Tylodelphys clavata*	97.14	117.65 (1–611)	114.29	97.14	13.21 (2–67)	12.83
Cestoda						
*Paradilepis scolecina*	45.71	3.88 (1–14)	1.77	91.43	11.28 (1–30)	10.31
Monogenea						
*Dactylogyrus sphyrna*	8.57	3.67 (1–5)	0.31	–	–	–
*Paradiplozoan homoin*	28.57	2.10 (1–9)	0.60	48.57	3.06 (1–18)	1.49
Nematoda						
*Contracaecum rudolphii*	–	–	–	8.57	1.00 (1)	0.09
*Philometra ovata*	11.43	1.25 (1–2)	0.14	–	–	–
Crustacea						
*Argulus foliaceus*	5.71	1.00 (1)	0.06	–	–	–
*Ergasilus sieboldi*	8.57	1.67 (1–3)	0.14	–	–	–
Ecological parameters						
Richness	9			6		
Shannon Index (H)	0.39			0.99		
Evenness (E)	0.18			0.55		
Simpson Index (D)	1.18			2.36		
Berger-Parker (BP)	0.92			0.50		

I, intensity; MA, mean abundance; MI, mean intensity; N, number of examined fishes; P, prevalence

### Statistical Analysis

**Fig. 1:**
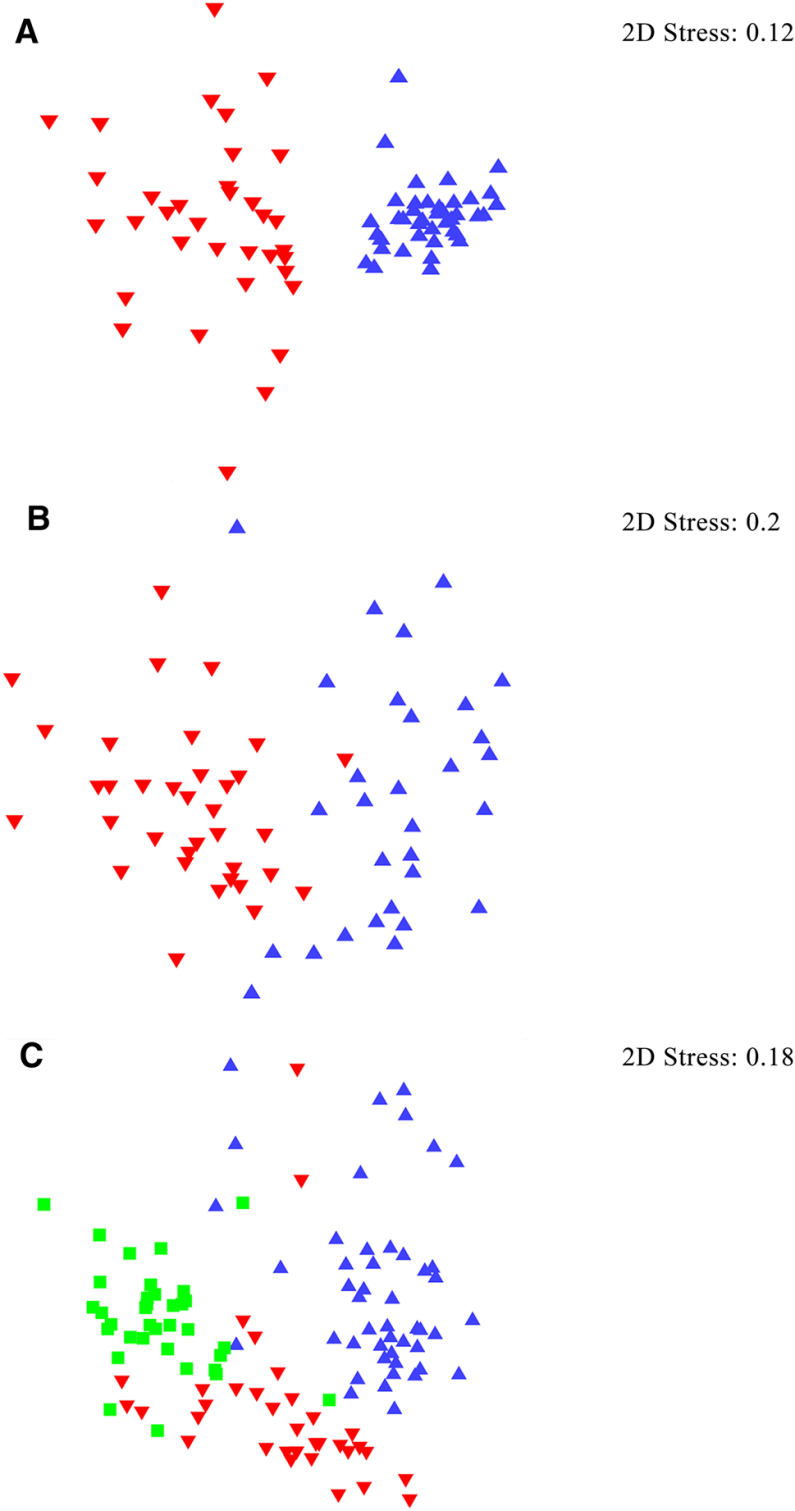
Non-metric multidimensional scaling (NMDS) plot showing separation of lakes based on the parasite communities of perch (**A**), bream (**B**) and roach (**C**) from different lakes from Mecklenburg-Western Pomerania, northern Germany by using Bray–Curtis similarity index (Symbols: Red triangle: Lake Malchin; Blue inverted triangle: Lake Tollense; Lime square: Lake Hohen Sprenz). For the purpose of statistical analysis, a single specimen of roach (Lake Malchin) was excluded due to presence of one individual parasite taxon (colour figure online)

A multivariate statistical analysis, Non-metric multidimensional scaling (NMDS) graph was performed in order to distinguish the habitats based on the parasite communities of the fish. Perch and bream showed separation of two habitats, Lake Tollense and Lake Malchin, based on their parasite communities. There is a clear separation of Lake Tollense and Lake Malchin based on perch parasite composition, while in bream, a few fish showed intermixing in both lakes (Fig. 1). ANOSIM results also supported this separation, with the values higher in perch (*R* = 0.73, *p* ≤ 0.001) compared to bream (*R* = 0.45, *p* ≤ 0.001).

In roach, separation was most distinct between Lake Tollense and Lake Hohen Sprenz, with lowest number of fishes mixing between these lakes (Fig. 1). There are more fishes overlapping between Lake Malchin and Lake Hohen Sprenz (Fig. 1). This is further supported by the value of ANOSIM (Global *R* = 0.57, *p* ≤ 0.001) between the lakes. The highest value was observed between Lake Tollense and Lake Hohen Sprenz (*R* = 0.75, *p* ≤ 0.001) while the lowest was between Lake Tollense and Lake Malchin (*R* = 0.43, *p* ≤ 0.001).

### SIMPER Analysis

SIMPER analysis was performed to identify the parasite species that were contributing most to the observed dissimilarity between the lakes for each fish species. For perch, a general dissimilarity of the parasite community of Lake Tollense and Lake Malchin is evident as both habitats create separated clusters (Fig. 1A). In perch, *B*. *luciopercae* and *Ichthyocotyluris variegatus* (Creplin, 1825) contributed the most to the dissimilarity between Lake Tollense and Lake Malchin (Contribution overall > 50%).

In bream, the dissimilarity of the parasite community from Lake Tollense and Lake Malchin was present, but not as prominent compared with perch. *Contracaecum rudolphii*, *D*. *spathacaeum*, Opecoelidae sp. and *Ichthyocotylurus platycephalus* (Creplin, 1825) were the most contributing parasite taxa to the dissimilarity between both lakes.

For roach, a certain grouping can be observed but is not as pronounced than in the other two fish species, as some fish are mixing. Comparing Lake Tollense and Lake Malchin roach, *T*. *clavata* and *D*. *sphyrna* contributed most to the dissimilarity. Same was evident for comparing Lake Tollense and Lake Malchin. Between Lake Malchin and Lake Hohen Sprenz, only *T*. *clavata* contributed almost 50% to their dissimilarity.

## Discussion

### Parasite Fauna of Lake Tollense

This study provides the first survey of the parasite community of three freshwater fish species, *P*. *fluviatilis*, *A*. *brama* and *R*. *rutilus* from Lake Tollense, northern Germany. The parasite fauna of Lake Tollense mostly consisted of autogenic species. Altogether, 32 parasite species were recorded, of which 24 species complete the entire life cycle in one ecosystem type (autogenic), while eight species had an allogenic life cycle. Of these allogenic species, seven were mature in fish-eating birds, whereas one digenean species*, Gorgoderina lufengensis* Gao and Zhang, 2014, uses frog as final host. In perch, the taxa *B. luciopercae*, *D*. *baeri*, *T*. *clavata* and *Camallanus* spp. were reported as core species, while monogeneans, acanthocephalans and crustaceans were classified as rare species (according to [27]). Two parasite taxa, *D. spathacaeum* and *C*. *rudolphii*, were considered as core species for bream. In roach, the core species were *A*. *limacoides*, *D. spathacaeum*, *T. clavata* and *D*. *sphyrna*.

This study provides a new locality record of *Rhipidocotyle campanula* (Dujardin, 1845) isolated from *P*. *fluviatilis*. This parasite species has been reported in *P. fluviatilis* from other countries such as Finland and Russia [10, 29]. A new host (*A. brama*) and locality record is described for *G. lufengensis*. This parasite taxon was first described from frog, *Paa yunnanensis* from China [30]. Same is true for *Lissorchis kritskyi* Barnhart & Powell, 1979 and *Prosorhynchoides borealis* Bartoli, Gibson & Bray, 2006 (in *R. rutilus*). *Lissorchis kritskyi* was first described from river carpsucker, *Carpiodes carpio* (Rafinesque, 1820) from USA [31], whereas *P*. *borealis* have been reported from angler fish, *Lophius piscatorius* (L.) from Iceland [32].

Similar to an earlier report by [33–36], digenean *D*. *baeri* inhabited perch, while only *D*. *spathacaeum* was identified in bream and roach. Similar findings have also been observed in previous studies in Germany. Moreover, *D. baeri* has also been reported from the Baltic Sea also in Mecklenburg Western-Pomerania in *Oncorhynchus mykiss* Walbaum, 1792 [37]. It is important to note that Unger *et al.* [38] studied the parasite fauna of *O*. *mykiss* and *Salvelinus alpinus* (L.) from a nearby aquaculture farm which uses the surface water of Lake Tollense. These authors reported only *D*. *spathacaeum* in the examined hosts, but not *D*. *baeri*. The absence of *D. baeri* in cyprinid hosts may be due to their resistance against this digenean taxon or could be related with differences in the biology and ecology of the first and second intermediate hosts (snails and fish, respectively).

### Comparison of the Parasite Fauna Between Water Bodies

Pikalov [18] investigated perch and bream from Lake Malchin and results revealed that these fish species have an almost similar composition of the parasite taxa compared with Lake Tollense, only exhibiting differences in their prevalence and abundance. In perch, the core species and generalist parasites *B. lucioperca*, *T. clavata* or *Camallanus* spp. were found in both habitats. In bream, the most common core and generalist parasite taxon in both lakes, Lake Tollense and Lake Malchin, was *C. rudolphii*.

In perch, prevalence and mean intensity of *I*. *variegatus* was much higher in Lake Malchin compared with Lake Tollense. Similarly, *I*. *platycephalus* was recorded from bream in Lake Malchin, but has not been found in Lake Tollense. The first intermediate host for both of these digenean species is a snail, *Valvata piscinalis* (Müller, 1774) [39]. The high abundance of *Ichthyocotylurus* spp. in Lake Malchin may be a result of the difference in abundance and seasonal distribution of snails as intermediate hosts in the lakes. Adverse climatic conditions, as it was shown by Mouthon and Daufresne [40] lead to a progressive decline and disappearance of the snail, *V. piscinalis*, in Saône River in France was related to higher water temperatures and a heatwave. In 2018, temperatures were exceptionally high in Mecklenburg-Western Pomerania, and this can be a reason for the observed low prevalence or absence of this digenean species in hosts inhabiting Lake Tollense. However, we have also examined perch (*n* = 21) in the following year from Lake Tollense and there was only a marginal increase in the prevalence of *I*. *variegatus*. Besides the temperature, there could be some other factors such as habitat characteristics. Jokela and Lively [41] showed that the prevalence of the larval trematode *Microphallus* sp. was significantly higher in shallow water habitats than in deep water. This indicates that habitat characteristics have a significant influence on the occurrence of digenean parasites. Lake Malchin is a shallow lake (average depth: 2.5 m). Therefore, we suggest that the combination of the factors temperature, lake characteristics, biological interactions, physical parameters and differences in distribution of the final and/or intermediate host were responsible for the observed variation in the occurrence and abundance of *Ichthyocotylurus* spp. between these two freshwater habitats.

For roach, the parasite fauna showed several differences in their prevalence and intensity between the studied lakes. In roach, only *D. spathaceum*, *T. clavata*, *P. homoion* and *P. scolecina* were constantly found in all lakes, but with differences in their occurrence and abundance. *Aspidogaster limacoides* was exclusively detected in Lake Tollense, but absent in both other habitats. Lake Tollense belongs to the Müritz freshwater system, an extensive natural lake and river system which represents an invasive species, the zebra mussel, *Dreissena polymorpha*, since the nineteenth century. The zebra mussel is a known definitive host for *A. limacoides* (see [42]). A study conducted by Prejs *et al.* [43] revealed that only roach with sizes of 16 cm and above feed on the zebra mussel (*D. polymorpha*). However, the average host size was comparatively higher in roach of Lake Tollense (standard length: about 21 cm) compared with Lake Malchin and Lake Hohen Sprenz (standard length: approximately 13 cm). On the other hand, Lake Hohen Sprenz is an isolated water body limiting species invasion and possibly also the presence of *A. limacoides*. Lake Malchin is connected to Lake Kummerow by the Peene, which drains into the Baltic Sea. It should also be kept in mind that *A. limacoides* does not show seasonal patterns as it was found throughout all seasons from Lake Tollense. Therefore, it can be concluded that the host size and habitat isolation were strong determinants for the presence or absence of *A. limacoides* in the sampled roach from different lakes.

### Diversity Parameters

It is notable that despite the differences in the lakes’ morphology and physico-chemical characteristics (isolation, topographical features and water quality), seasonality (years and seasons) and host attributes (host size and weight), parasite diversity parameters did not differ significantly between perch and bream.

Unlike perch and bream, roach showed variation in the diversity indices at the three localities. The most diverse parasite community was observed in Lake Tollense followed by Lake Malchin and the more isolated Lake Hohen Sprenz. This can be explained by the fact that Lake Tollense (17.9 km^2^) has the largest area of all studied lakes and belongs to the second largest river and lake system in Germany, the Müritz. Previous studies support our results regarding the lake size and the number of parasite species, for instance, Kennedy [44] found significant a correlation between species richness and lake size in brown trout (*Salmo trutta* L.), but not in Arctic charr, *S. alpinus* [45]. Our finding showed a positive association between the lake size and the species richness in roach.

The highest Shannon Wiener Index was detected from Lake Tollense followed by Lake Hohen Sprenz and Lake Malchin. Despite of having a smaller area, Lake Hohen Sprenz showed a higher Shannon index than Lake Malchin and the average host size was almost similar in both habitats. This suggests that the Shannon diversity is not always positively associated with the habitat size as well as host size. Similarly, a higher evenness was seen in Lake Hohen Sprenz compared with Lake Malchin. Previously, Salzmann *et al.* [15] found lower values for both these indices (Shannon index and evenness) in Lake Dörpfeld and stated that it was related with a small surface area and lack of fish stocking. However, this study does not support the explanation that low diversity and evenness in lakes refers to a small surface area as given by Salzmann *et al.* [15]. However, Salzmann *et al.* [15] studied artificial and anthropogenic strongly influenced freshwater systems in North-west Germany while the herewith sampled lakes are of ice age origin and surrounded by more pristine environments. Another possible reason for the observed differences in the diversity indices between these three lakes could be associated with their different trophic status of water bodies, e.g. Lake Tollense is a less disturbed habitat and thus, has a higher diversity while both other lakes are much more disturbed and hence, have low parasite species diversity. We could demonstrate that the differences of the diversity values for roach are mainly driven by the ectoparasites (three species in Lake Tollense, two species in Lake Malchin with low abundance and one species in Lake Hohen Sprenz).

### Statistical Analysis

The parasite composition of perch, bream and roach was visualised by using NMDS, which showed distinct separation of the lakes with some minor overlapping depending on the fish species. All three fish species revealed separation of the three habitats. This was further supported by the result of ANOSIM which showed that there was a highly significant variation between lakes based on the parasite communities of studied fish species. This supports the notion by Salzmann *et al.* [15] that it is possible to distinguish freshwater ecosystems based on the parasite fauna of roach. However, identification of the concise reasons is more difficult and also requires parasitological analyses of other freshwater species.

## Conclusion

It could be shown that based on the multivariate statistical analysis, NMDS composition of the parasite fauna of three sampled fish species differed between the studied lakes and therefore can be used to differentiate and characterise these natural freshwater habitats in Mecklenburg-Western Pomerania. Despite distinctly different trophic statuses of the three lakes, other differences in their topographical features and sampling time (between years and in seasons) and host attributes (e.g. host size) contributed to this result. Moreover, the diversity parameters were almost similar in perch and bream between two lakes, Lake Tollense and Lake Malchin, but not in roach where both diversity indices as well as the NMDS revealed a clear separation of habitats by this host. This suggests that in more natural, less influenced freshwater habitats the combination of two methods, in our case the diversity and NMDS, were required to evaluate the observed differences. The application of biological indicators must be strongly associated with concise differences in habitats (see [14]) if fish parasites can be used as relevant biological indicators for freshwater ecosystems in future. Further studies should focus on the investigation of the best suitable freshwater fish species for characterising distinct ecosystems and habitats and thus generating evaluable and comparable data.

## Data Availability

All data published within this text.
